# Psycho-social impact of stillbirths on women and their families in Tamil Nadu, India – a qualitative study

**DOI:** 10.1186/s12884-018-1742-0

**Published:** 2018-04-20

**Authors:** Vijayaprasad Gopichandran, Sudharshini Subramaniam, Maria Jusler Kalsingh

**Affiliations:** 1 Department of Community Medicine, ESIC Medical College & PGIMSR, KK Nagar, Chennai, Tamil Nadu 600078 India; 2 NODAL Point, Chennai, Tamil Nadu 600033 India; 3 Institute of Community Medicine, Madras Medical College, Chennai, Tamil Nadu 600003 India

**Keywords:** Stillbirth, Psycho-social impact, Grief, Guilt, Health system, Coping

## Abstract

**Background:**

Stillbirth has serious psycho-social consequences on the parents and on the family. The psychological impact of stillbirth is strongly influenced by the social and cultural context. There is very scarce information on this from the Indian context. This qualitative study was conducted to understand the psycho-social impact, aggravating factors, coping styles and health system response to stillbirths.

**Methods:**

A qualitative study was conducted using in-depth interviews with mothers who experienced stillbirth in the past 1 year and their families. A total of 8 women and two health care providers were interviewed by trained interviewers. The interviews were transcribed into the local language and thematic analysis was performed by the researchers retaining the transcripts in the local language. Themes were identified, and a conceptual framework was developed.

**Results:**

Women who experienced stillbirths suffered from serious forms of grief and guilt. These emotions were aggravated by the insensitive health system, health care providers, friends, and neighbours, as well as strained marital relationship and financial burdens. The women and their families were disturbed by the ‘suddenness’ of the stillbirth and frantically searched for the cause. They were frustrated when they couldn’t find the cause and blamed various people in their lives. The women and their families perceived poor quality of services provided in the health system and reported that the health care providers were inconsiderate and insensitive. On the other hand, the health care providers reported that they were over-worked, and the health facilities were under-staffed. The community health workers reported that they felt caught in the crossfire between the health facility staff and the family who suffered the stillbirth. The women reported several coping mechanisms including isolation, immersion in work, placing maternal love on other children, the anticipation of next pregnancy and religiosity.

**Conclusion:**

Stillbirth is a major cause of psycho-social morbidity. Health systems should be responsive to the psycho-social needs of women who suffer stillbirths and their families.

**Electronic supplementary material:**

The online version of this article (10.1186/s12884-018-1742-0) contains supplementary material, which is available to authorized users.

## Background

Stillbirth, defined as the birth of a baby at or after 28 weeks of gestation, without any signs of life, is one of the most neglected maternal and child health problems. In the year 2015, worldwide the stillbirth rate was 18.4 per 1000 total births [1]. An estimated 2.6 million stillbirths occur annually in the world, out of which 98% occur in low and middle-income countries. More than half of them are due to preventable causes [1]. The Global Every New-born Action Plan (ENAP), was launched in June 2014 during the 67th World Health Assembly, to advance the Global Strategy for Women’s and Children’s Health. The ENAP sets forth a vision of a world that has eliminated preventable new-born deaths and stillbirths. India, a signatory to this plan, brought out the Indian New-born Action Plan with the goal of a single digit stillbirth rate [2]. However, stillbirths were never addressed as a problem in the Millennium Development Goals and they do not feature in the Sustainable Development Goals either [3]. Given this major problem of stillbirths and the silence of global policies on it, the Lancet published a series on stillbirths in January 2016 [4]. One of the major problems of stillbirths is its significant association with psycho-social morbidity for the mother and the family. The economic and psycho-social burden of stillbirths significantly impacts the health of the population in low and middle-income countries [4]. A systematic review of qualitative studies has highlighted that a woman suffering stillbirth experiences grief, guilt, pain, and stigma [5]. Not much is documented on the experiences of Indian women on suffering a stillbirth. In settings like India with a large section of the population living in lower socioeconomic conditions, the psycho-social impact of stillbirth is unique [6, 7]. Much less is known about the influences of the health system in aggravating or mitigating these psycho-social consequences. Therefore, there is a need to explore the social, emotional and psychological impact of stillbirths on women and their families in the Indian context.

## Methods

### Study setting

The study was conducted in and around Chennai, the capital city of the state of Tamil Nadu, in southern India. It is a metropolitan city with a population of about 7.1 million. Because of its expanding boundaries and a huge number of development projects in its suburbs, the city has been rechristened as the Greater Chennai Corporation, which is home to the third largest population of internal migrant workers in India [8]. The public health services in Chennai are delivered by the Corporation through its Corporation Health Centres. As Greater Chennai includes some parts of the districts of Kancheepuram and Tiruvallur, these areas are served by the Directorate of Public Health and Preventive Medicine, Government of Tamil Nadu. Thus, the participants of the study were served both by the urban and rural health systems even though they lived in a geographically proximate area. Tamil Nadu has some of the best indicators for maternal and child health in the country. For the year 2014, according to the civil registration system of the Government of Tamil Nadu, the birth rate in Chennai was 17 per 1000, and death rate was 9 per 1000. Infant mortality rate of Tamil Nadu was 20 per 1000 live births. Stillbirth rate in Tamil Nadu in the same year was 6.8 per 1000 live births [9]. The institutional delivery rate in Chennai is nearly universal and therefore the reports of infant mortality and stillbirth rates are reliable.

### Study design

The study was conducted using qualitative research methods. To understand the experiences of stillbirth from the perspective of women and their families, in depth interviews were conducted among women who experienced stillbirths within the past 1 year and their families. There was an open-ended and iteratively modified checklist which evolved with each interview. The items included in the checklist were – the experience of stillbirth, feelings, and emotions related to the experience, support received, coping strategies, social impact, impact on family and meaning attributed to the experience. The checklist can be found as a Additional file 1.

### Sampling

Participants were women who experienced a stillbirth within the past 1 year. This time constraint was placed to limit problems with recall of events associated with the stillbirth. Even though having a sample of women who had experienced stillbirth more than a year ago could have provided valuable insights into the women’s experiences, in the long run, the balance between obtaining experiences which are fresh in their memory and obtaining long term experiences had to be maintained and hence the constraint on time was placed. They were identified with the help of the community health workers in the local area. The community health workers spoke to the mothers and their family and sought permission for the researchers to visit and interview them. The community health worker accompanied the researchers to the home of the woman who experienced the stillbirth and introduced the researchers to the family.

Initially, six women were interviewed. They were the only available women in the study area who had experienced stillbirth in the past 1 year. Data saturation was observed as most of the themes were repetitive. After a time gap, two more women who suffered stillbirths were identified by the health workers. Their interviews were conducted to confirm the data saturation and the interviews were stopped at eight. Two interviews, one each with a community health worker and a hospital duty nurse were conducted to triangulate the data obtained from the families. None of the participants who were approached refused to participate in the study.

Out of the eight women interviewed, all of them had delivered in public health facilities, a majority of seven of them had first pregnancies and only one of them had a living son. The cause for stillbirth was abruptio placentae in one of the women, severe pregnancy induced hypertension in another, cervical incompetence in one, and the exact cause of stillbirth was not known in the remaining five. Irrespective of the cause of the stillbirth, the psychological and social experiences of these women were similar.

### Interviews

The interviews were conducted in the privacy of the home of the women. In all the interviews one other family member was present during the interview. In the cultural context of Tamil Nadu, it is customary to not leave a young post-partum woman alone with strangers. Therefore, a family member was always present during the interviews. In some instances, it was the mother of the woman, in some, it was the mother in law and in some the husband. Moreover, the accompanying person was an active participant in the interviews and they also shared perspectives of the family. Therefore, rather than the deep insights of experience of the woman, the interviews gathered insights of experiences of the women and their families. However, it is likely that the relationship dynamics between the family members influenced the interviews. Each woman and her family member were interviewed only once. Each interview lasted about 45 min to a maximum of 2 h. During the interviews, intensive notes were taken. These notes were transcribed on the same day as the interview into elaborate transcripts. No audio-visual recording of the interviews was conducted to preserve the cultural and emotional sensitivity of the situation. Copies of the transcripts were shared with the women through emails wherever possible and they were encouraged to comment on the fidelity of transcription. None of the women responded to these emails with any suggestions for changing the transcript. A post-interview debriefing was conducted after each interview among the research team. These field debriefing notes were also documented and used for analysis. The interviews were conducted and transcribed in the Tamil language.

### Research team and reflexivity

The research team included all the three authors, who were present during all the interviews. All three are formally trained in qualitative interview techniques. While one of the researchers conducted the interview, the other two took extensive notes and prepared the transcripts and field notes. One of the researchers is a woman and in the interviews that she conducted, the mothers and their families were more participative and responsive. Therefore, she conducted more interviews than the other two researchers who are men. Two of the researchers are physicians and this identity was used by the community health workers to approach the mothers and their families to seek permission for the interviews. This was probably the major reason for a predominant focus of the interviews on health system faults and difficulties faced in the health facilities. None of the research participants knew the researchers ahead of time. Adequate time was spent before the start of the interview in establishing rapport with the women and their family members.

### Analysis

The data was analysed by two of the researchers using thematic analysis method. The coding and analysis were performed in English while retaining the original transcripts in Tamil to preserve the fidelity of the narrative. After identifying the quotes, they were finally translated into English to present them in the report. Open coding was performed in the Microsoft Excel Spreadsheet. Coding was grounded in the data. New codes were generated based on detailed reading and interpretation of the transcripts. The coding performed by both the researchers was verified and validated by the first researcher. The themes were then developed based on the codes. After developing the themes, a conceptual framework was developed. Appropriate quotes were identified to substantiate the themes and subthemes.

### Ethical considerations

The study was reviewed and approved by the Ethics Committee of the Madras Medical College, Chennai. Written informed consent was obtained from all the participants before the interview. The anonymity of participants was maintained during analysis and reporting to protect their confidentiality.

## Results

**Fig. 1:**
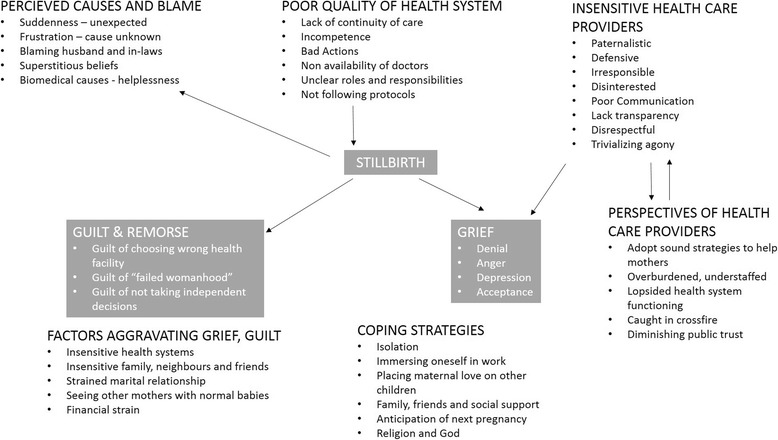
Conceptual model of psycho-social impact of stillbirth. This figure illustrates the conceptual model of psycho-social impact of stillbirths on women and their families. The main psychological impacts are grief and guilt. The perceived poor quality of the health system and insensitivity of health providers were attributed by the women as causes for the stillbirth. The conceptual model also explains the factors aggravating the grief and guilt, coping styles and the perspective of the health care providers

The stillbirth scenario of each of the interviewed women is described in Table 1. Several important themes emerged from the interviews. Important among them were bad experiences due to insensitive attitudes of health care providers, and poor quality of services in the health facilities. The women and the families who suffered stillbirth were searching for causes for the stillbirth and blamed various people. The major psychological consequences were grief and guilt. The grief and guilt were aggravated by several factors. The health care providers shared their perspectives on stillbirth. These themes are elaborated below. Some verbatim quotes are presented in this section. Some more verbatim quotes are presented in the Additional file 2. The conceptual framework emerging from these themes is illustrated in Fig. 1.

**Table 1:** Stillbirth Scenario in each of the interviewed women

S. No	Gestational Age at Stillbirth (weeks)	Place of Stillbirth	Parity	Antepartum / Intrapartum	Cause of Stillbirth
1	38	Primary Health Centre	Primi	Antepartum	Not determined
2	32	Primary Health Centre	5th Para	Intrapartum	Cervical Incompetence
3	36	Primary Health Centre	Primi	Intrapartum	Not determined
4	38	Primary Health Centre	First Para (previous abortion)	Antepartum	Not determined
5	36	Primary Health Centre	Primi	Antepartum	Abruptio placentae
6	38	Primary Health Centre	Primi	Antepartum	Severe Pregnancy Induced Hypertension
7	36	Primary Health Centre	Primi	Intrapartum	Not determined
8	38	Primary Health Centre	Primi	Intrapartum	Not determined

### Insensitive attitudes of health care providers

The mothers and relatives felt that insensitive attitude of the doctors, nurses and health care providers added to the pain of stillbirth. They described this insensitive attitude of the health care providers as:

i. Paternalistic decision making on behalf of the patients
ii. Defensive attitude that they did not cause the stillbirth and they are not to blame. According to a family, in one instance they even lied to protect their own name.

*“to protect themselves, the nurse came outside the labour room and told everybody waiting outside that the mother’s life is in danger. They told this lie to protect themselves because now if the baby died they will be relieved that at least the mother is saved” – husband of a mother who suffered a stillbirth*

iii. Not taking responsibility for the health of the mother and child. One of the parents said,

*“the doctor said that in government hospital they can provide only these services, if we want more, we should go to private hospitals” – mother in law of a woman who had a stillbirth*

iv. Disinterested and apathetic attitude, which was explained by one of the women,

*“when I was admitted to the labour ward, I had pain all night. In the morning during duty change time, the sister on duty for the morning shift did not come. So, they had to call another nurse who was not on duty that day. It was a Sunday morning and she was very much irritated. She showed her irritation on me” – a mother who suffered a stillbirth*

v. Lack of good communication with the mothers,

*“They never told us or explained to us the reason for the stillbirth. We were also extremely upset and so we couldn’t ask.” – a mother who had a stillbirth*

vi. Disrespectful attitude

*“The nurses attending on me in the labour ward were disrespectful. They scolded me and verbally abused me. What happened to me is over, I hope this never happens to any other person” – a mother who suffered a stillbirth*

vii. Trivializing attitude, reducing the stillbirth to a mere statistic.

### Poor quality of health system and services

The mothers and their families also blamed the health system of poor quality of services. They described:

i. Lack of continuity of care with frequent change of health care providers in shift work, leading to higher incidence of mistakes due to poor communication between staff.
ii. Incompetent health care providers,

*“I myself could feel that my baby is not moving, and my baby is dead. But they could not diagnose it and could not explain anything to me.” – a mother who suffered a stillbirth.*

iii. Bad practices followed by health care providers were narrated by some of the mothers.

*“two nurses and then an aayah (housekeeping staff) – all three of them repeatedly applied pressure on my abdomen to force the delivery of the dead baby. They repeatedly put their hands inside my private parts and because of that my private parts got swollen up. Because of the pain I was biting my lips and my lips were also swollen up. I couldn’t take the way they handled me. I was screaming in pain” – a mother who suffered a stillbirth.*

iv. Doctor was not available in many of the primary health centres.
v. The families felt that the health care providers were not performing their assigned duties. The nurses did the doctors’ job, the housekeeping staff did the nurse’s job and so on.

*“None of the staff do their assigned roles and duties. Ayah (housekeeping staff) is doing the work of the nurse and nurse is doing the work of the doctor. Therefore, finally, nobody is answerable to patients.” – husband of a woman who suffered a stillbirth*

vi. In some instances, the families perceived that the health care providers did not follow due protocol when they were admitted to labour ward and this lead to the death of their baby.

*“I was suffering from pain all night and I did not feel the baby movements. If they had immediately done a scan, I think they could have saved the baby. They did not do the scan.” – husband of a mother who suffered a stillbirth*

### Search for cause and blame

i. In most of the interviews the families expressed shock over the ‘suddenness’ of the stillbirth. It was unexpected in all the cases. One of the mothers said,

*“I developed labour pains in the 9* ^*th*^ *month. Immediately after the onset of pains, I went to the hospital. The pain was present all through the night. In the morning, suddenly the sister checked and told me that the baby’s heart beat has stopped. I was shocked.”- mother who suffered stillbirth*

ii. Many mothers and families expressed a sense of frustration due to inability to identify the cause of the stillbirth. In some instances, this translated to a general anxiety about the wellbeing of the next pregnancy.

*“it was all so sudden, we did not know why the baby died. I am worried what will happen to the next baby. But nobody seems to know the cause” – mother in law of a mother who suffered a stillbirth*

iii. In some of the interviews, the women blamed their husbands and in-laws for not caring for them and their pregnancy. This was largely reflective of the patriarchal tendencies in traditional households where women continued to perform strenuous manual work even during pregnancy. There was also a tendency in the society for the in-laws to blame the parents of the woman who suffered a stillbirth. This was also expressed in some of the interviews.

*“My anger is completely focused on my husband. If he had only paid proper attention to me during pregnancy, I would have had a live baby” – a mother who suffered a stillbirth*

iv. In one of the interviews, an elderly woman held some superstitious beliefs as the cause for the stillbirth. The belief was related to unfulfilled duty to the dead in the family.
v. Some of the educated women attributed the stillbirth to biomedical causes. They were accepting of the explanation provided to them by health care providers and saw stillbirth as a purely biomedical process.

*“I was much older than other women when I got pregnant. This was because I took treatment for a long time for getting pregnant. My husband had low sperm count and he also took treatment. It is because of my age, and weakness in both me and my husband that my baby died” – a mother who suffered a stillbirth.*

### Guilt and remorse

i. The women who experienced insensitivity and lack of competence in the public health system reported guilt in choosing the health facility. They felt that if they had not hesitated to spend money out of pocket in a private health facility to have a safe delivery, they would have delivered a healthy baby.

*“the biggest mistake I did was going to that hospital (primary health centre) for my delivery. If only I had gone to a private hospital, I would have had a normal baby. My guilt is killing me, if only I had chosen the right hospital…” – a mother who suffered a stillbirth*

ii. Some women perceived their inability to give birth to a live child as a failure of their womanhood.

*“I felt very guilty. I felt worthless that I am not even able to give birth to a normal healthy child.” – a mother who suffered a stillbirth*

iii. One of the women who suffered stillbirth felt very guilty for not taking a firm stand and an independent decision about her delivery.

*“when I got pain I called my husband to the office. He came in his two-wheeler. I climbed on to it and we both went to the hospital. I should have taken a firm decision to go to private hospital. I should not have gone to the primary health centre. I am feeling guilty for not taking a firm decision” – a mother who suffered a stillbirth*

### Grief

The various stages of grief as proposed by Elizabeth Kubler Ross and David Kessler were evident from the interviews [10].

i. Denial **–** Some mothers expressed a sense of denial and disbelief.

*“I never believed that my baby died. It took me some time to realize the truth” – a mother who suffered a stillbirth*

ii. Anger **–** Families who had crossed a few weeks after the event expressed anger. Their anger was directed mainly on the health system, and the health care providers. In some cases, the anger was also directed at the in-laws.

*“the death of our baby was totally because of the negligence of the doctor and sisters in the hospital. we are very angry with them. I went and shouted at the staff of the hospital immediately” – mother in law of a woman who suffered a stillbirth*

iii. Depression **–** All the families expressed a stage of depression that was prolonged. There were significant depression and distress in all the family members who participated in the interviews. They narrated that the depression extended to the entire family.

*“nowadays I am unable to concentrate on any work. I am always crying. I am not even able to engage in a conversation with anyone.” – a mother who suffered a stillbirth*

iv. Acceptance **–** Some of the families who had crossed many weeks following the stillbirth, expressed a level of acceptance of the event.

*“I have now accepted the fact that my baby has died. I have to become normal for the sake of the family.” – a mother who suffered a stillbirth*

### Factors aggravating grief and guilt

i. Admission of the women in the general post-natal ward where there are other mothers with normal and healthy babies aggravated the grief.

*“After the stillbirth, I was admitted in the general ward where other women who had delivery were also admitted. I used to feel very sad when I saw the other mothers with healthy babies” – a mother who suffered a stillbirth*

ii. Insensitive comments and remarks by family members, friends and neighbours upset them. Some women isolated themselves to protect themselves from their insensitive remarks.

*“our neighbours called us murderers. That made us feel very bad” – a mother who suffered a stillbirth*

iii. Some women mentioned that their marital relationship was strained because of the stillbirth. This worsened their grief.

*“After the stillbirth, there is a lot of fights between my husband and me. We don’t have a cordial relationship. I think only having another baby will set it right.” – a woman who suffered a stillbirth*

iv. When the mothers who suffered stillbirth saw other mothers with normal children, they felt very sad and felt a longing to have their own babies. Therefore, they avoided putting themselves in such situations where they must interact with other mothers.

*“when I see other mothers in my area with babies, I feel very sad. Nowadays I don’t go to buy milk. My friends who have healthy babies will come to buy milk at the same time and if I see them I feel like crying” – a mother who suffered a stillbirth*

v. Another external factor which compounded the grief in some women was a financial strain. Borrowing money for treatment and difficulties in repaying the loans was perceived as a major factor worsening the grief.

*“because of the repeated blood tests, scans and check-up, we had to incur a lot of expenditure. We had to take a lot of loans. Already, the stillbirth made us feel bad. Above this, the loan and financial burden made us feel worse” – a mother who suffered a stillbirth*

### Coping strategies

i. Women who suffered stillbirth, initially tend to isolate themselves from the society because of guilt, grief and shame. They also perceived a sense of stigma of failed womanhood. Culturally they perceived themselves to be inauspicious and isolated themselves from participating in social functions.

*“I don’t even go to temples. I don’t go anywhere. I stay inside the house. I know if I see people, they will feel sad for me and that will make me feel sad.” – a mother who suffered a stillbirth*

ii. For many women immersing themselves in their work helped them forget the pain of the stillbirth.

*“the best way to overcome my sadness is to get immersed in household work. I started doing all cooking, cleaning and household work immediately. This helps me forget the pain a little bit” – a mother who suffered a stillbirth*

iii. Some women expressed their maternal love towards other children in the household such as children of siblings.

*“I take care of my sister-in-law’s children. On Sundays, we sit and watch television together and play together. This helps me forget my stillbirth pain. I also help in preparing them for school daily and help them with their homework” – a mother who suffered a stillbirth*

iv. Some of the women who were interviewed, after an initial phase of isolation and self-pity, sought the support of friends and family.

*“my neighbours and relatives were very supportive. They spoke to me nicely and helped me overcome my sadness.” – a woman who suffered a stillbirth*

v. Many women said that they wanted to get pregnant again and give birth to a healthy baby. They said that the anticipation of a normal delivery and a healthy baby keeps them going.

*“I have hope that I can have a normal baby next time. I need to know what all I have to do to give birth to a normal baby”- a mother who suffered a stillbirth*

vi. Some women felt that belief in God and visiting the temple helped them cope with the grief of stillbirth. Other women mentioned that they directed their anger against God and stopped going to the temple.

*“Going to the temple helps me to cope with my sadness.” – mother who suffered stillbirth*

### Perspectives of health care providers

#### Strategies adopted to support mothers suffering stillbirth

The health providers including community health workers and hospital staff mentioned that stillbirth is a traumatic event and should not be disclosed to the mother immediately. So, they concealed the information from the mother till she is discharged from the hospital. They told her that the baby is unwell. They advise the family to share the information with the mother only after she becomes stable. The community health worker also mentioned that she advised and counseled the mothers. She told them not to worry. The health workers encourage them that they will have a normal baby next time.

#### Overburdened and understaffed health system

The health care providers mentioned anecdotally that the incidence of stillbirths has increased in the recent times. They attributed this increase in stillbirths at the primary health care facility to increasing rates of complicated pregnancies, increasing age of women who became pregnant, difficulties in providing antenatal services in urban slums and migrant colonies where there were overcrowding and language difficulties and increase in the load of women who have started to come to primary health care services for delivery. They also mentioned that they are over-worked and understaffed and therefore work under high levels of pressure.

“*I am working as a village health nurse (Community Health Worker) for the past 27 years. In those days, we had innocent women who were younger as our clients. Nowadays women get pregnant much later. This leads to complications. Not only this, the women and their families are well informed. They know everything and ask a lot of questions. Because of this, we are seeing a lot of complications” – a Community Health Worker.*

#### Lopsided health system functioning

The health care providers felt that development and improvements in the health system were lopsided and top heavy. While there are many staff, equipment, and facilities in the tertiary care centres, primary level care is still under-staffed and under-equipped. Even if equipment and infrastructure are made available, there is a lack of people who can manage them.

*“we used to have a very skilled doctor in this hospital previously. Now madam has retired. They have posted a young male doctor in our primary health centre. He is good but does not know much. Therefore, mistakes have increased. We have scan machine, but the doctor who does scan moves from this centre to the other centre nearby and is not always available. So even though we are a 24-hour delivery facility, we don’t have enough people to work or to operate the equipment. How can we say this and explain to the patients?” – a Duty Nurse in a Primary Health Centre*

#### Community health workers caught in the cross fire

The community level health workers felt a sense of being caught in the crossfire between the women who suffered stillbirth, their families and the staff in the health facilities. They complained that stillbirths and other mistakes were happening in the health facilities. However, for every case of stillbirth, neonatal death or maternal death, the public health system holds the community health worker accountable. Moreover, the community health workers are also answerable to the women who suffer the stillbirth and their families.

*“They say a drum gets beatings from both sides. Like that, we get abused by the higher officials as well as from the community. when something bad like a stillbirth or a neonatal death happens, the higher authorities also question and scold us. The families in the community also scold us. We must take abuse from both sides. Sometimes the nurses in the primary health centre will treat the patients badly. For this also, we will get scolded in the community” – a Village Health Nurse*

#### Diminishing public trust in health providers

The health providers also felt that the overall trust in the public health system was diminishing. They attributed this to increased awareness among the people, increased ability to ask questions, increased access to information and knowledge.

## Discussion

This qualitative exploration of the psycho-social impact of stillbirth on women in Tamil Nadu, south India, showed that women suffered from grief, guilt, and remorse because of a stillbirth experience. The women and their families perceived stillbirth to be a very sudden, unexpected, confusing and frustrating experience, as the exact cause was not explained to them clearly. They attributed various explanations including superstitions, biomedical explanations and blamed various persons in their lives for the occurrence. Overall there was a strong narrative of dissatisfaction and anger towards the health system. There was also a perception that the health care providers were insensitive, negligent and disrespectful of mothers who suffered stillbirths. Several factors affected the grief and guilt that the women felt. These included unresponsive health system, insensitive family, friends and neighbours, strained marital relationship, social stressors and financial strain. The women had both positive and negative coping mechanisms including, isolation, support from friends and family and placing their maternal love on other children. From the perspective of the health care providers who cared for women who suffered stillbirths, it was seen that they were overworked, the health facilities understaffed and often the community health workers were caught in the crossfire between the health facilities and the families. There was also a narrative of diminishing public trust in the health system.

### Quality of health system

One of the strongest narratives in this study was that of the poor quality of the health system. Most mothers and their families mentioned that they did everything within their limits to care for the pregnancy, and the stillbirth is mainly attributable to the poor quality of care provided by the health system. The narrative focused on describing the insensitive and lackadaisical attitude of the health care providers. There were also complaints of bad clinical practices like performing unscientific treatments like applying fundal pressure for delivery, performing unnecessary vaginal examinations without aseptic precautions, delay in diagnosis, delay in identifying complications, and delay in referrals.

It could be argued that these are complaints of dissatisfied and disgruntled clients of the health system. But, it is unlikely to be the case. The complaints can be triangulated with the interviews with the health care providers. The health care providers also mentioned that they did whatever was the best within their capacity. They complained of overburdened health facilities with understaffing. They also complained that the development and functioning of the health system were lopsided, with tertiary care facilities getting more funding, equipment, personnel compared to primary care facilities. Moreover, the health care providers in the community also complained of being caught in the crossfire between the health facilities providing poor quality care and disgruntled patients and their families. There is a need to immediately improve the quality of services provided at the health facilities with an overall primary care approach. In urban and peri-urban areas in India, health care falls through the gaps and large proportions of the population fall outside the safety net of the public health system [11, 12]. Closely associated with the high level of dissatisfaction with the quality of care in the health system and blaming of the health system was the frustration and search for causes for the stillbirth, which most of these families had.

### Search for cause of stillbirth

The whole experience of stillbirth was surreal for the parents and the family. They were confused and frustrated. The entire experience was so ‘sudden’ and unexpected that it compounded the frustration. They did not understand the cause of the stillbirth. The search for a cause, the frustration arising from this, and the consequent blaming of various people for the stillbirth were common in most of the interviews. These feelings have been reported in previous studies [13–15]. However, the frantic search for causes and blaming took some unique cultural flavours in this study. In some families where there were strained relationships with mother in law and sister in law, they were blamed for the stillbirth. Several superstitious beliefs like, dissatisfied spirits of deceased elderly people wandering in the house and intake of inappropriate foods were also attributed as causes. The suddenness of the whole event took the families by shock. In several interviews, the overwhelming feeling was frustration, acceptance of a weak biomedical explanation for the stillbirth and helplessness. Another observation is that irrespective of whether the stillbirth was intra-partum or ante-partum, the women did not have any difference in their experiences. The potential preventability of an intra-partum stillbirth was not known to many of these women.

### Grief

Several previous studies have described grief in parents who suffer stillbirths. The grief has been described as ambiguous and lacking a legitimate space for expression [13]. The disenfranchised grief was attributed to lack of personhood to the unborn foetus [5]. A type of complicated grief which is a combination of disbelief, yearning, anger and depression has also been described [16]. Along with various types of grief associated with stillbirth, another psychological morbidity has also been described such as depression, anxiety, agoraphobia, social phobia, guilt, and suicidal ideations [5]. A study from Chhattisgarh, India showed that perinatal grief in Indian women is characterized by sadness, unhappy feeling, shame, guilt, dishonour, lowered self-worth, fear and yearning for a child [17, 18]. This study also revealed that the health care providers did not address the mother’s grief appropriately. The health workers tended to trivialize the mother’s and family’s grief. Parental grief not being legitimized was also reported in a systematic review on economic and psychological consequences of stillbirth [4]. In a study from Africa, it was reported that in certain cultures the stillborn is not given the status of personhood and so grieving the stillborn is not socially sanctioned [19]. In the present study, grief was expressed in the stages described by Kubler-Ross and Kessler namely, denial, anger, depression and acceptance [10]. The grief was legitimized and shared by the entire family and society. There are reports of family, friends, neighbours and relatives being supportive of the loss and grief. The cultural context in which this study is set, seems to respect and legitimize the grief of women who suffer stillbirths and their families. The legitimization of the grief may be a more recent phenomenon, with reduction of family size in the state of Tamil Nadu and greater value placed on the life of each child and personhood attributed to them. The other possibility for legitimizing of grief in this context is that ultrasound scans, visualization of the foetus and hearing the foetal heart sounds have become almost universal, thus attributing personhood to the unborn baby. The attribution of personhood to the unborn foetus helps legitimize the grief of the parents and the family.

### Guilt

Guilt has also been described previously as a major psychological consequence of stillbirth. In a detailed qualitative study of Muslim women, it was found that guilt was a dominant emotional impact of stillbirth [20]. In another interpretative phenomenological exploration, a sense of questioning of the self and excessive guilt was reported as a major emotion in mothers who suffered stillbirth during their first pregnancy [21]. In a large-scale quantitative survey conducted in Bangladesh among women who suffered stillbirth, the prevalence of depression was as high as 43% and there was also a major proportion of women who felt guilty [22]. The guilt of failed womanhood or failed motherhood was described in a previous phenomenological study of women who suffered a stillbirth [15]. In an interesting systematic review of the psychological impact of stillbirth, self-blame and guilt were described as major findings [5]. In the present study guilt was articulated as blaming oneself for choosing the wrong health facility, where good quality care was not available for her, guilt of failed “womanhood” or failed “motherhood” and guilt of not taking an independent decision about care seeking. In the predominantly peri-urban and urban areas around Chennai, where the interviews were conducted, half the women who were interviewed were well educated and were employed. Therefore, there was a possibility for these women to take independent and well-informed decisions. However, the strong patriarchal attitudes in the society, and the dominant decision-making powers of elders in the family (e.g. mother in law) often impaired her independent decision making. It can be seen in this study that women felt guilty for succumbing to these patriarchal and family pressures and not taking their own independent decisions. Failure of “motherhood” or “womanhood” as seen in this study has also been expressed in several previous studies. The guilt of “body failure” has also been described in a previous study [23]. In the cultural context of this study, becoming a mother is perceived as the hallmark of womanhood. Giving birth to dead babies and infertility are highly stigmatized and are viewed as a failure of womanhood. This was perceived as a guilty feeling by these women. Moreover, stillbirth strongly threatened the ‘moral status’ of a woman. If a woman is not capable of bearing healthy children, she is considered as deviating from her moral responsibility [24].

### Factors aggravating grief and guilt

This study also revealed several factors in the socio-cultural environment of the mothers which aggravate the feelings of grief and guilt. Insensitive health facilities which place the mothers who have suffered a stillbirth alongside the cheerful and happy mothers who have a live baby in the hospital ward, was aggravating the grief and sorrow. Similar aggravation has been reported in previous studies in the west [13]. In the public health system in the setting of the study, there is a limitation in the number of beds. Lack of space, beds and resources puts pressure on the hospital staff to place mothers who suffered stillbirth alongside normal postpartum mothers. There is a need to be more sensitive to the feelings of these mothers and probably negotiate space for them within the general ward or the antenatal ward. The women felt that the pity felt by well-meaning friends and relatives aggravated their sorrow. One of the strategies reportedly followed by the community health workers was to counsel them about future pregnancies and how to take care of themselves for the future. The women said that they found such well-meaning advice insensitive. In a previous study, comments about the next baby and future care, were reported to be insensitive and trivializing the loss of the stillbirth [13]. Rather than pity, counseling, advice and consolation, the women should be provided support and their intense grief acknowledged appropriately. This would help them heal better. Strained marital relationships were reported by the women in this study. Previous studies in the western context have revealed intense grief due to perinatal loss leading to divorce, difficulties in relationships and difficulties in sexual intimacy between spouses [5, 25]. The difference in the grieving styles between the fathers and mothers and lack of attention to the father’s grief were factors adversely affecting the marital relationships [22, 26, 27]. In a study of the psychological impact of stillbirth on fathers, it was seen that the fathers resorted to negative coping strategies such as alcohol abuse, illegal drugs and extra marital relationships [28]. These factors could not be explored in the study, but it is not uncommon to have such psychological impacts on the father in the context of the study, which would in turn adversely impact the marital relationship. The mothers who suffered stillbirth avoided encountering other women who had live babies, as seeing them aggravated their grief. This has been reported previously in various socio-cultural contexts [15, 29]. Coming in contact with other mothers with live babies produced resentment, jealousy, reminded them of their dead baby and made them feel bad. Yet another important aggravating factor relevant in the context of resource-deprived communities is a financial burden. Especially, given that good quality care is lacking in the public health system and good quality care can only be obtained through spending out of pocket in private health facilities, events like stillbirth pushed several families into indebtedness. This worsened the grief and guilt further in resource-limited families.

### Coping strategies

The women and their families had both positive and negative coping styles. It was seen that the women isolated themselves for a period from the society to protect themselves from the aggravation of their grief. They also immersed themselves in their work to cope with the pain of stillbirth. The literature on coping with death and grief shows that men tend to cope by immersing themselves in their work [30]. In our study, the women seemed to use this coping style to overcome their grief. A previous study highlighted the gender difference in coping styles following the loss of a baby to stillbirth. The study showed that men tend to worry, use the support of friends and relatives and ignore the issue, whereas women sought spiritual and religious support, used wishful thinking about the future and sought support from others who suffered a similar grief [31]. In the present study, the women used religion and God, support from friends and family and wishful thinking about future pregnancy as coping strategies as shown in the other study. Another coping mechanism used by the women in the present study which is unique to this social context is placing maternal love and care on other children in the family.

In summary, stillbirth was a life-changing event in most of the interviewed families. There was a strong sense of dissatisfaction with the health system and health care providers. While the families frantically searched for the cause of the stillbirth, they blamed various people in their lives as well as superstitious factors and ultimately accepted weak biomedical explanations with a sense of helplessness. Grief and guilt were the overwhelming psychological impact of the stillbirth in the families. These feelings were aggravated by factors in the health system, the family and the society. To overcome the grief and guilt women adopted various positive coping strategies.

From this discussion, it emerges that there is a need for a deep understanding of the psychological emotional impact among women who suffer stillbirth and their families. The health system should be strengthened and sensitized to the specific needs of these women. Hospital and health facilities should have policies that are sensitive to the psychological and emotional needs of women who suffer stillbirths. Health care providers should be provided sensitization in addressing the emotions of women who suffer stillbirths and their families. Women who suffer stillbirths require information, counseling, acknowledgment of their grief, support from health system, families, society. In the bargain, the husband and the family should not be neglected, and they should also be embraced in the protective net.

This study of the psycho-social impact of stillbirth in a low resource urban setting in India, is the first to explore the phenomenon of stillbirth in detail using qualitative methods. It provides deep insights into the experiences, emotions, psychological problems, and coping mechanisms of these women. The study identified the various types of insensitive attitudes of the health care providers as described by the women who suffered stillbirth and their families. These attitudes can be addressed through focused training of the providers to create a sensitive work force to provide care for these women. There are certain important limitations in this study. The most important is that the study did not attempt to understand the process of grieving of a stillbirth in the local cultural context. While the emotional aspects of grief have been discussed, the grieving rituals, the cultural meaning of stillbirth and the notions associated with it have not been explored. These explorations would have provided a deeper insight into the legitimization of the grief of stillbirth. A deeper study of the grieving process of the fathers could not be performed and this is a main limitation of the study. Triangulation with interviews of neighbours, community members, religious leaders etc., could have provided a complete social understanding of stillbirth. A phenomenological exploration would have given deeper insights into the “experience” of suffering a stillbirth. Future studies should focus on exploring the phenomenon. Tamil Nadu as explained previously, has very low stillbirth rate in the country. If the same study had been done in a setting with more number of stillbirths, a different picture of the psycho-social impact might have emerged. There is a need to explore the issue from a broader pan-Indian perspective.

## Conclusion

In conclusion, stillbirth, though a biomedical problem, is a significant psycho-social phenomenon that has potential to change the life of a family. Therefore, care for mothers who suffer stillbirth should include a thorough attention to the psycho-socio-cultural aspects of the event. Health systems should be sensitive to such psycho-social needs of women who suffer stillbirths and their families.

## Additional files

Additional file 1: In depth interview Checklist. This file contains the checklist used for conducting the in-depth interviews. (DOCX 16 kb)

Additional file 2: Table: Verbatim quotes supporting the themes. This file contains the table which describes the various themes and some verbatim quotes from the interviews supporting the themes. (DOCX 23 kb)

## Abbreviation

ENAP: Every New-born Action Plan
