# Supplementary material for: Psycho-social impact of stillbirths on women and their families in Tamil Nadu, India – a qualitative study
Source: BMC Pregnancy Childbirth. 2018 Apr 20;18:109. doi: 10.1186/s12884-018-1742-0 (PMC5910626; doi:10.1186/s12884-018-1742-0)
Supplement: Supplementary file 1 — In depth interview Checklist. This file contains the checklist used for conducting the in-depth interviews. (DOCX 16 kb) [file 12884_2018_1742_MOESM1_ESM.docx]

**Social, emotional and psychological impact of pregnancy loss – a socio-ecological analysis**

**In depth interview checklist**

| **S. No.** | **Main Question** | **Probe Questions** |
| --- | --- | --- |
| **Emotional and Psychological Experiences** | | |
| 1 | What do you think about motherhood experience for a woman? (தாய்மை) | Is it essential?  Why is it important for a woman to become a mother?  What are the expectations about motherhood a woman has when she is pregnant?  How has all this been affected because of the death of your baby? |
| 2 | It must have been a really painful experience losing your child during delivery. Could you please tell me about it? | Can you describe the events of the day of your delivery?  When and how did you know that the baby had died? |
| 3 | How did you feel when you came to know that your baby had died? (தங்கள் உணர்வுகள் என்ன?) | What was the immediate feeling?  Have you had a similar feeling before for any other incident? Can you describe the feeling? |
| 4 | Can you tell me some of the emotions and feelings that went through your mind during your post-delivery phase after you lost the baby? | Did you feel angry? On whom was your anger directed? (கோபம்) Why?  Did you feel hopeless? Why? (நம்பிக்கையற்ற நிலை)  Did you blame yourself? (பழி)  Did you blame anybody for this problem? Whom did you blame? Why?  Did you think you could have prevented the death of the child?  Did you feel that the life is unfair? (நீதி இல்லை)  Did you ask “why me”? |

| 5 | How did these emotions affect your life? (இந்த உணர்வுகள் தங்கள் வாழ்கையை எப்படி பாதித்தது?) | What did you do when you felt these emotions?  How did these emotions affect your everyday life? (அன்றாட வாழ்கை)  How did these emotions affect your relationships? (உறவுகள்)  Were you worried about what others would think?  How did these emotions affect your work / job? |
| --- | --- | --- |
| 6 | How did you manage these feelings? (இந்த உணர்வுகளை எதிர்கொள்ள என்ன செய்தீர்கள்?) | What did you do to overcome these sad feelings?  How long did it take?  Did you adopt any methods to overcome these feelings? |
| **Social issues** | | |
| S. No. | Main Question | Probe Questions |
| 1 | I understood how painful the experience of losing your child was for you. Apart from your own personal experiences you would have also faced social problems because you lost your child. Can you tell me about the social problems that you faced? (சமூக ரீதியான பிரச்சனைகள்) | How did your family respond to the death of your baby?  How did your husband react?  How did your parents react?  How did your in-laws react?  Did they blame you? Blame God? Resort to spiritual support? Withdrew from you? (தங்களிடமிருந்து விலகி சென்றார்களா?) Resort to other coping mechanisms like drinking / smoking etc? |
| 2 | How did your neighbours / society react to the loss of your baby? | How did your neighbours react to the loss of your baby? Did they sympathize? (பரிதாபப் பட்டார்களா?) Blame you? Isolate you? (தள்ளி வைத்தார்களா?) Talk ill about you? (தவறாக பேசினார்களா?) |

| 3 | Did you face any kind of isolation / discrimination in the society because your child died? (தனிமை படுத்தப்படுதல்/ பாகுபாடு பார்த்தல்) | Were you able to participate in social occasions like birthdays / weddings / baby showers?  Were you able to go to places of interests like temple / park / beach / mall etc?  Did people talk negatively about your child’s death?  Did anybody blame you / your family?  Were you considered inauspicious / unlucky? (அபசகுனம்) |
| --- | --- | --- |
| 4 | Did your family (husband / parents / in laws) face any isolation / discrimination because your child died? | Were your family able to participate in social occasions like wedding / birthdays / baby showers?  Were they able to go to places of interest like temples / park / beach / mall etc?  Were they considered inauspicious or unlucky? |
| 5 | How are you managing the social issues that you are facing because of the loss of our child? | Are you isolating yourself?  Have you stopped doing activities that you used to enjoy doing before?  Have you found alternative social activities which minimize the stigma and discrimination? (களங்கம், பாகுபாடு இல்லாத வேறு செயல்கள்) |

| **Social Support** | | |
| --- | --- | --- |
| S. No. | Main Question | Probe Questions |
| 1 | The delivery time when you lost your baby, as you have described before, was the most painful time. Can you tell me what are the kinds of support that you received during this time? | What financial support did you receive? (பொருளாதார உதவி)  What physical support (transport / care taking / food) support did you receive?  What emotional support did you receive?  What advice / intellectual support did you receive? |
| 2 | Tell me about the availability and accessibility of these supports? | Did you have to search and look out for these supports or were they easily accessible?  Did you have any troubles other than access issues to get these supports? |
| **Health System Support** | | |
| 3 | Did you find the health system (hospital / nursing home / public health delivery system) supportive? | Were the doctors sensitive to your condition (loss of your baby) and treat you with sensitivity? (உணர்ச்சிகளை மதித்து நடந்துகொள்ளுதல்)  How did the nurses treat you? Were they sensitive to your condition?  How did other health care providers treat you?  Did the doctors and nurses address your emotional distress? (உணர்வுபூர்வமான துயரம்)  Did the doctors and nurses answer your doubts and concerns in a sensitive manner? |
| **General Questions** | | |
| 4 | Thank you very much for sharing your experiences of going through a very bad phase of your life. From your experience of losing a child, what do you think are the important supportive measures that a woman undergoing the loss of a baby should receive? | From the health system?  From the society?  From the family?  How should this sensitization be achieved?  Do you think the government should play a role in this? |
