# Supplementary material for: Psycho-social impact of stillbirths on women and their families in Tamil Nadu, India – a qualitative study
Source: BMC Pregnancy Childbirth. 2018 Apr 20;18:109. doi: 10.1186/s12884-018-1742-0 (PMC5910626; doi:10.1186/s12884-018-1742-0)
Supplement: Supplementary file 2 — Table: Verbatim quotes supporting the themes. This file contains the table which describes the various themes and some verbatim quotes from the interviews supporting the themes. (DOCX 23 kb) [file 12884_2018_1742_MOESM2_ESM.docx]

Supplementary Table: Verbatim quotes supporting the themes

| Main Theme | Sub-theme | Verbatim Quote |
| --- | --- | --- |
| Insensitive attitude of health care providers | Paternalistic attitude | “The hospital sister (nurse) told me that this one surviving child is enough for me. I should not keep trying for another baby. I should take proper care of this one.” – mother who had recurrent stillbirths. |
|  | Defensive attitude | “When I had the stillbirth I was admitted in the hospital for 15 days. The doctors and sisters never explained or spoke anything to me or my family. They explained only when they wanted some signature from us. As soon as we signed the forms, they went away.” – mother who had stillbirth |
|  |  | “Even after diagnosing that my baby was dead, they refused to refer me to the tertiary hospital. They were afraid that the doctors and nurses in the tertiary hospital will blame them for treating me very badly. They delayed referring and it lead to problems” – mother who suffered a stillbirth |
|  |  | “when we showed the report of the scan done on the previous day which showed that the baby was healthy, the doctor asked us to tear it and throw it on the face of the doctor who did the scan. He said it was a false report” – husband of woman who had stillbirth |
|  | Irresponsible attitude | “The nurse told us that saving the mother was itself good enough, so what if they couldn’t save the baby” – mother in law of woman who had stillbirth |
|  | Disinterested in work | “Immediately after my stillbirth, I was on a urinary catheter. I couldn’t even get up and walk. Whenever I requested a ward sister (nurse) to help me get up, she never responded.” – mother who had recurrent stillbirths |
|  | Poor communication | “The senior doctor will scold the junior doctor in front of us in the wards. They spoke in English. We never understood what they are saying. I knew that they were talking about my treatment. But they never explained to me about what is being said.” – mother who suffered a stillbirth |
|  | Lack of transparency | “when she was inside undergoing the labour pains, we were standing outside in a helpless state. They never told us that there was a problem. They kept saying that everything is going on well and normal delivery will happen soon.” – husband of a mother who suffered a stillbirth |
|  | Trivializing attitude | “the nurse came and told sorry to me after the baby died. She said that this kind of death of a baby is not very rare and one in 1000 will be a stillbirth. But how can we accept that kind of a statement. Out baby is not just another number” – mother who suffered a stillbirth |
|  |  | “for the staff in the hospital, delivery of a dead baby is just a routine job. But for me it is a life changing event. Why can’t they understand that?” – mother who suffered a stillbirth |
| Poor Quality of Health System and Services | Lack of continuity of care | “the doctors in the hospital keep changing during shift change. we don’t know who is the doctor in charge of our treatment. We don’t know whether the new doctor has been informed about our treatment. Similarly nurses also keep changing shifts. Sometimes we don’t know whether a person is a doctor or a nurse. They never communicate with us. We feel if one doctor took care of us, we can ask them questions and we will be more comfortable”- husband of a mother who suffered a stillbirth |
|  | Bad practices followed by health care providers | “Because of the way they ill-treated her, the discharge from her private parts became yellowish in colour. After some time pus started oozing from the vagina. The baby was also dead and the mother also suffered from infection. Then we had to go away to a private hospital, get admitted and treated for the infection” – mother in law of a woman who suffered stillbirth |
|  | Non availability of doctor | “when we got admitted in the hospital, there was only a nurse on duty. She checked us and told us that everything is normal and delivery will happen soon. But there was no doctor” – mother who suffered a stillbirth |
|  | Not following due protocol | “They made us wait in the hospital unnecessarily. As soon as they identified that there is a problem they should have referred us. Unnecessary delay lead to death of my baby” – mother who suffered a stillbirth. |
| Search for cause and blame | Suddenness | “when I asked the doctor about the cause for the stillbirth, they kept deferring the answer. They kept saying come tomorrow, I will tell you, come next day I will tell you and so on. Ultimately I never understood the reason for this” – mother who suffered a stillbirth |
|  |  | “I don’t understand why this happened. The doctors and nurses are not able to explain the cause for the death of my baby.” – mother who suffered a stillbirth |
|  | Blaming husband and inlaws of negligence | “in my house, my parents in law did not care for me well. If the same stillbirth had occurred when I was living in my mother’s place, they would have blamed my parents for it. Now, it has happened here because they did not care for me. If only they had cared for me well, I would have given birth to a live child” – mother who suffered a stillbirth |
|  |  | “On that day I could not feel the movements of the baby, I also started having low back pain. I immediately told my husband. He told his mother. Then we also told his sister. They all asked me to ignore it as it may be false pain. They asked me to drink warm water and sleep. If I had immediately sought help at that time, now my baby would have been alive” – mother who suffered a stillbirth |
|  | Superstitious beliefs | “when she was pregnant, an elderly woman passed away near our house. Instead of doing an elaborate 16-day death ritual for that old lady, they did only 8-day rituals. Therefore, the spirit of the old lady was dissatisfied and it took revenge on the family by causing this stillbirth.” – mother of a woman who suffered stillbirth. |
|  |  | “we used to live in a haunted house previously. I got pregnant when I was living there. I think that is the reason for this baby’s death” – mother who suffered stillbirth |
|  | Biomedical causes | “the nurse in the hospital told us that the baby’s growth was inadequate and so the baby died. What can we do about it? We should have cared enough and seen that the baby’s growth is adequate” – mother in law of a woman who suffered a stillbirth |
|  |  | “On the day of the delivery, I was sweating profusely. My hands and feet were swollen. My abdomen was hard like a stone. I thought this must be because of high blood pressure. I also thought my baby must have died.” – mother who suffered a stillbirth |
| Grief | Denial | “when they did the scan and told me that the baby was dead, I did not believe them. I was feeling very numb.” – mother who suffered a stillbirth |
|  | Anger | “nowadays I am feeling very angry at my mother. I shout at her all the time.” – mother who suffered stillbirth |
|  |  | “I am feeling angry at God. How can this happen to us?” – father who suffered a stillbirth |
|  | Depression | “my parents are devastated by the death of our child. when some relatives come to visit us, my mother immediately starts crying.” – mother who suffered a stillbirth |
|  |  | “I was so upset by the death of my baby that I felt that I should also die.” – mother who suffered a stillbirth |
|  |  | “The death of this baby has completely shattered both our families. It is a major reason for sadness. We will never be able to overcome this loss” – father who suffered a stillbirth |
|  |  | “the dreams and aspirations of our families were completely shattered by the death of the baby, especially because this is the first baby of the next generation in the family” – mother who suffered stillbirth |
| Factors aggravating grief and guilt | Insensitive health care providers and health system | “in the big hospital, immediately after delivery, they put me in the general ward where other mothers who delivered their babies were also admitted. When a woman with a normal delivery came, and since there were no empty beds, they asked me to shift to the floor. I felt very bad” – mother who suffered stillbirth |
|  | Insensitive family, friends and neighbours | “my sister also had a normal delivery around the same time I had my stillbirth. So I felt very bad to stay in my mother’s house, as it was considered inauspicious. So I stayed here in my mother in law’s home. I felt very sad” – mother who suffered stillbirth |
|  |  | “nowadays I am not able to go to any functions or festivals. This is because when my relatives see me, they feel sorry and talk with pity. I hate that feeling. It makes me feel sad” – mother who suffered stillbirth. |
|  | Seeing other mothers with healthy children | “in my street, 4 of us were pregnant at the same time. We were all friends and we went for all check-up together. Now when I see them with their babies, I feel very sad and feel like crying” – mother who suffered stillbirth |
| Coping Strategies | Isolation | “women like me who have lost their babies are considered inauspicious. They (society) will talk badly if we even touch their normal babies. So I do not go for any social functions” – mother who suffered stillbirth |
|  |  | “initially I isolated myself from all my friends, neighbours and relatives. Nowadays I have started going to temple alone” – woman who suffered stillbirth |
|  | Immersing self in work | “I specifically asked for evening shift at my work place. So, I will do household work all day long. Then I will go to office in the evening and return home around 2 AM. I will feel exhausted and go to sleep. This is my method of forgetting the pain of stillbirth” – mother who suffered stillbirth |
|  | Placing maternal love on other children | “my brother’s son is living in the house downstairs. I like to take care of him. He calls me mother. This helps me to overcome my sadness” – mother who suffered stillbirth |
|  | Family, friends and social support | “my biggest support system is my office colleagues and friends. They came home after I was discharged and visited me frequently. We used to hang out and keep chatting and joking. That helped me cope” – woman who suffered stillbirth |
|  |  | “our biggest support is our friends. They helped us both financially, physically as well as emotionally. When everybody in the society was blaming us, they stood by us and supported us” – father who suffered stillbirth |
|  | Religion and God | “I got so angry with God, that I couldn’t go to temple anymore. I stopped even praying to God.” – mother who suffered stillbirth |
| Perspectives of health care providers | Strategies adopted to support mothers suffering stillbirth | “If a stillbirth happens to a woman, I visit her home. After all the immediate grief period is over, I counsel her. I tell her that she will have a healthy baby soon and advise her on eating healthy foods. I also tell her to come for regular check-up for next pregnancy and encourage her” – Community Health Worker |
|  | Overburdened and understaffed health system | “In my long years of service as a village health nurse, I have seen only one stillbirth so far. That too in a woman from a migrant colony. It was 5th pregnancy for her. She did not even register with me, as she did not even know that she was pregnant. One day she came to the sub centre with abdominal pain. When I examined, I saw that it was breech presentation. I rushed her to the tertiary care centre. There the baby was stillborn.” – Village Health Nurse |
|  |  | “we are overworked, they give us a lot of responsibilities and at the same time they also question us if we don’t perform some of the duties perfectly. I am in charge of 18,000 people in my area. We are actually supposed to care for only 5000 population as per norms. It is very difficult to visit all the women, know about their pregnancy and date of delivery etc. In addition to this, they also make us do other projects like immunization coverage drive, dengue prevention drive and so on. How can we perform optimally?” – Village Health Nurse |
|  | Lopsided health system functioning | “We are located close to the tertiary care centres. The tertiary care centres have excellent facilities and lot of doctors and nurses to take care of the women. So we regularly send the patients there. But the relatives of the patients get angry with us for referring them. They do not understand that we don’t have enough facilities or skilled people to handle the complications” – Nurse in a primary health centre |
|  | Diminishing public trust in health providers | “In those days, the women used to trust everything that we told them. they used to take their iron and folic acid tablets regularly, take tetanus injections and come for check-up. Now women are more educated. They ask a lot of questions. They don’t believe everything we say. They doubt us.” – Village Health Nurse |
